# VJ21.089The subcompartmented oxphosomic model of the phosphorylating system organization in mitochondria

**DOI:** 10.18699/VJ21.089

**Published:** 2021-11

**Authors:** I.V. Ukolova

**Affiliations:** Сибирский институт физиологии и биохимии растений Сибирского отделения Российской академии наук, Иркутск, Россия

**Keywords:** system of oxidative phosphorylation, mitochondria, oxphosome, models of the OXPHOS organization, supercomplexes, система окислительного фосфорилирования, митохондрии, оксфосома, модели организации ОКСФОС, суперкомплексы

## Abstract

The oxidative phosphorylation (OXPHOS) system of mitochondria supports all the vitally important energy-consuming processes in eukaryotic cells, providing them with energy in the form of ATP. OXPHOS enzymes (complexes I–V) are located in the inner mitochondrial membrane, mainly in the cristae subcompartment. At present, there is a large body of data evidencing that the respiratory complexes I, III2 and IV under in vivo conditions can physically interact with each other in diverse stoichiometry, thereby forming supercomplexes. Despite active accumulation of knowledge about the structure of the main supercomplexes of the OXPHOS system, its physical and functional organization in vivo remains unclear. Contemporary models of the OXPHOS system’s organization in the inner membrane of mitochondria are contradictory and presume the existence of either highly organized respiratory strings, or, by contrast, a set of randomly dispersed respiratory supercomplexes and complexes. Furthermore, it is assumed that ATP-synthase (complex V) does not form associations with respiratory enzymes and operates autonomously. Our latest data obtained on mitochondria of etiolated shoots of pea evidence the possibility of physical association between the respiratory supercomplexes and dimeric ATP-synthase. These data have allowed us to reconsider the contemporary concept of the phosphorylation system organization and propose a new subcompartmented oxphosomic model. According to this model, a substantial number of the OXPHOS complexes form oxphosomes, which in a def inite stoichiometry include complexes I–V and are located predominantly in the cristae subcompartment of mitochondria in the form of highly organized strings or patches. These suprastructures represent “mini-factories” for ATP production. It is assumed that such an organization (1) contributes to increasing the eff iciency of the OXPHOS system operation, (2) involves new levels of activity regulation, and (3) may determine the inner membrane morphology to some extent. The review discusses the proposed model in detail. For a better understanding of the matter, the history of development of concepts concerning the OXPHOS organization with the emphasis on recent contemporary models is brief ly considered. The principal experimental data accumulated over the past 40 years, which conf irm the validity of the oxphosomic hypothesis, are also provided.

## Abstract (ru)

Система окислительного фосфорилирования (ОКСФОС) митохондрий поддерживает все жизненно важные энергозатратные процессы в клетках эукариот, обеспечивая их энергией в форме АТФ. Ферменты ОКСФОС (комплексы I–V) локализуются во внутренней мембране митохондрий, преимущественно в кристном субкомпартменте. К настоящему времени получен значительный объем данных, указывающих на то, что дыхательные комплексы I, III2 и IV в условиях in vivo могут физически взаимодействовать друг с другом в различной стехиометрии, образуя суперкомплексы. Несмотря на активное накопление знаний о структуре основных суперкомплексов системы ОКСФОС, ее физическая и функциональная организация in vivo остается неясной. Современные модели организации ОКСФОС во внутренней мембране митохондрий противоречивы и предполагают существование либо высокоорганизованных дыхательных цепочек, либо, наоборот, набора случайно расположенных дыхательных суперкомплексов и комплексов. При этом предполагается, что АТФ-синтаза (комплекс V) не образует ассоциаций с дыхательными ферментами и работает автономно. Наши последние данные, полученные на митохондриях этиолированных побегов гороха, указывают на возможность физической ассоциации дыхательных суперкомплексов и димерной АТФ-синтазы. Эта информация позволила пересмотреть существующие представления об организации фосфорилирующей системы и предложить новую субкомпартментационную оксфосомную модель. Согласно новой модели, значительная часть комплексов ОКСФОС формирует оксфосомы, которые в определенной стехиометрии включают комплексы I–V и располагаются преимущественно в кристном субкомпартменте митохондрий в виде высокоорганизованных цепочек или «патчей», представляющих собой «мини-фабрики» по производству АТФ. Предполагается, что такая организация способствует увеличению эффективности работы системы ОКСФОС; открывает новые возможности для регуляции ее активности и в той или иной степени может определять морфологию внутренней мембраны митохондрий. В обзоре подробно обсуждается предлагаемая модель. Для лучшего понимания вопроса кратко рассмотрена история развития представлений об организации системы ОКСФОС с акцентом на современные модели, а также приведены накопленные за последние сорок лет основные экспериментальные данные, подтверждающие обоснованность оксфосомной гипотезы.

## Introduction

The system of oxidative phosphorylation (OXPHOS ) of mitochondria is the main source of energy generated in the form of ATP, which is necessary for maintaining all vitally important metabolic processes taking place in the cells of aerobic eukaryotic organisms. The OXPHOS enzymes are localized in the inner membrane of mitochondria and include five functional complexes I–V, each representing a complexly organized molecular machine: complex I (NADH-dehydrogenase), complex II (succinate dehydrogenase), complex III (cytochrome-bc1-complex), complex IV (cytochrome с oxidase) and complex V (ATP-synthase). The four first enzymes form the respiratory chain and are sequentially involved in the process of transfer of electrons from the oxidizable substrate upon the molecular oxygen. This process in complexes I, III and IV is coupled with translocation of protons across the inner mitochondrial membrane, as a result of which an electrochemical proton gradient is formed, which is used by ATP-synthase for ATP synthesis. Furthermore, mobile electron carriers, ubiquinone and cytochrome c (Enríquez, 2016), as well as the translocators of adenine nucleotides and inorganic phosphate coupled with ATP-synthase are attributed to the OXPHOS system (Luzikov, 2009).

The components of the energy transformation system make up the bulk of proteins of the inner mitochondrial membrane and, according to various sources, occupy from half to two-thirds of its hydrophobic volume (Vonck, 2012; Schlame, 2021). To date, a lot of data evidencing the higherordered organization of the OXPHOS enzymes in vivo have been accumulated. Existence of respiratory supercomplexes, which include respiratory complexes I, dimer III2, and IV in various stoichiometry, as well as the presence of oligomeric ATP-synthase in the inner mitochondrial membrane have been proven (Vonck, 2012; Chaban et al., 2014). It is believed that such a compact supramolecular organization gives the possibility of avoiding nonspecific aggregation of enzymes and deformation of the lipid bilayer (Guigas, Weiss, 2016), increases the efficiency of respiration, and protects the cell from oxidative stress (Lenaz, Genova, 2012).

However, despite the fact that the structure of the main supercomplexes has been sufficiently studied, the physical and functional organization of the OXPHOS system in vivo remains unknown and still is a matter of controversy. Activeinterest in this issue may be explained by the fact that correct understanding of native organization of the energy system in mitochondria not only opens new opportunities for further development of mitochondriology, but also determines new ways of solving such vitally important problems of mankind as therapy of diseases associated with mitochondrial dysfunctions. 

To date, possible variants of the arrangement of supercomplexes in the inner mitochondrial membrane are considered in contemporary models of the phosphorylating system organization, which are sometimes contradictory and assume existence of either highly organized respiratory strings, or, vice versa, respiratory supercomplexes and complexes freely diffusing in the membrane plane. Furthermore, it is assumed that ATP-synthase does not form associations with the respiratory enzymes and functions autonomously.

**Fig. 1.**
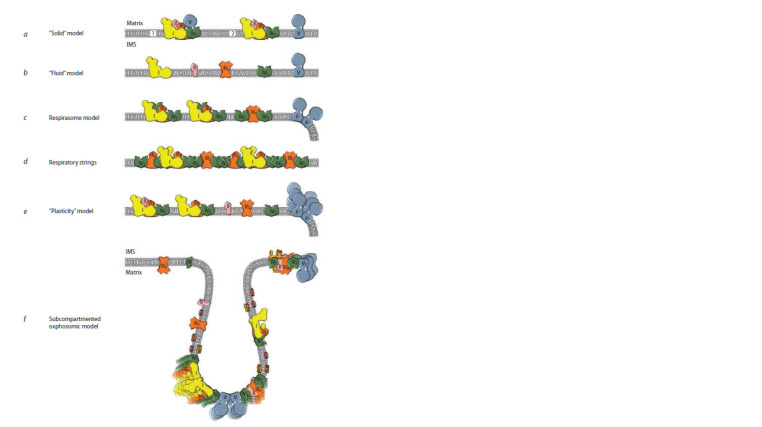
Development of ideas related to the conception of the OXPHOS system organization in mitochondria, from the initial “solid” model to the proposed subcompartmented oxphosomic one. The interpretation of the models is presented taking into account recent data on the structure of OXPHOS complexes of mammals (a–e) and plants (f ). According to the literary data, all the principal models (with the exception of model f ) are given for mammalian mitochondria. Denotations: (1) initial and (2) later “solid” models; matrix and IMS – matrix and, respectively, intermembrane space of mitochondria; complexes I, II, III2, IV and IV2 of the respiratory chain and ATP-synthase (complex V) are shown in yellow, pink, orange, green and blue, respectively. It is shown on more recent schemes that ATP-synthase dimers bend the membrane and, thereby, are involved in the formation of cristae (c, e, f ). Description of the models can be found in the text. The respirasome model (c) is given in accordance with the schemes of H. Schägger (Schägger, Pfeiffer, 2000; Schägger, 2002); it ref lects the principal postulate of the model, which determines the ratio of the large supercomplex I1III2IV4 and the small supercomplex III2IV4 as 2:1. The model of respiratory strings (d) is shown according to the scheme of H. Schägger and I. Wittig (Wittig, Schägger, 2009), but, unlike the representation in the original, the f igure shows a side view (in the plane of the membrane). There are other variants of the respiratory strings (Bultema et al., 2009; Miranda-Astudillo et al., 2018). In the oxphosomic model (f ), developed for the mitochondrial OXPHOS system of etiolated pea shoots (Ukolova et al., 2020), in addition to the main OXPHOS complexes, there are also freely located alternative enzymes, which indicates a more complex organization of the phosphorylating system in plants.

Recently, on the basis of our data obtained with the use of mitochondria from pea shoots, a subcompartmented oxphosomic model of organization of the phosphorylating system has been proposed (Ukolova et al., 2020). This model, in contrast to existing ones, postulates that a substantial part of population of the respiratory supercomplexes interacts with dimeric ATPsynthase in vivo, thereby forming the oxphosomes, which are located mainly in the cristae subcompartment of mitochondria as highly organized strings or patches (Fig. 1, f ). Such an organization is expected to substantially elevate efficiency and involve additional levels of control over the operation of the OXPHOS system. This new model is discussed below. For the purpose of better understanding the issue and assessing the validity of the model, the review provides a brief history of evolution of the views on the organization of the OXPHOS system, and also literary data maintaining the oxphosomic hypothesis.

## A brief history of development of ideas on the mitochondrial OXPHOS system organization in vivo

The physical integrity of the respiratory chain (i. e. the unity of its components) was assumed long ago in publications by D. Keilin and his co-authors in the 1930s–1940s (Keilin, 1930; Keilin, Hartree, 1939, 1949). For a long time, it was believed that all the enzymes of the respiratory chain interact stably to form “respiratory assemblies” (Chance, Williams, 1956; Lehninger, 1959). Such an aggregation state of the respiratory chain was called “solid” (Lehninger, 1959; Rich, 1981) (see Fig. 1, a). As new data became available, the “solid” model was replaced with the “fluid” one (Hackenbrock et al., 1986) (see Fig. 1, b). This model excluded the physical association of OXPHOS components and postulated that all the redox components involved in electron transfer and the proteins required for synthesis of ATP represent “independent lateral diffusants” that interact in course of multiple collisions

Despite the existence of a large amount of data confirming the validity of the “fluid” model, facts indicating the existence in vivo of (i) associations of respiratory chain complexes and (ii) oligomeric ATP-synthase continued to accumulate. The year 2000 became a turning point, when H. Schägger and his colleagues (Schägger, Pfeiffer, 2000), using the method of blue native electrophoresis (BN-PAGE) (elaborated by them earlier), obtained convincing evidence of physical interaction between the respiratory complexes leading to formation of supercomplexes. Thus they actually updated and returned the “solid” model, proposing the respirasome model (see Fig. 1, c). According to this model, the found supercomplexes are the “building blocks” that “can interact to form a network of respiratory chain complexes that may be called a respirasome”. Later, the authors (Schägger, 2002) began to call a separate supercomplex, comprising complexes I, III2, and IV the respirasome, because this superstructure could independently “respire”, i. e. provide for the entire cycle of electron transfer from the oxidized substrate to the molecular oxygen. As a result, this term has taken root and is currently used in the literature in this context.

## Contemporary understanding of the energy system organization in mitochondria

Phylogenetic conservation of organization of OXPHOS components

With the advent of BN-PAGE and further successful combination of this method with cryoelectron microscopy, in-gel enzyme activity assays, and other methods, the study of supramolecular organization of the OXPHOS system in mitochondria of various organisms has reached a principally new level. Further investigations of this system in organelles from mammalian, plant, fungi, yeast, algae, and some protozoa revealed a similar composition of the supercomplexes (Krause et al., 2004; Chaban et al., 2014). All supramolecular associations of the OXPHOS components, obtained as a result of solubilization of mitochondria with the use of mild detergents, may be subdivided into four main groups: (1) supercomplex I1III2; (2) supercomplexes III2IV1–2; (3) respirasomes I1III2IV1–4; and (4) dimeric ATP-synthase. In some species, other respiratory supercomplexes of distinct compositions and stoichiometry were found (see Ukolova et al., 2020). Dimers of ATP-synthases in vivo assemble into long oligomeric rows at the cristae rims (Kühlbrandt, 2019). There are convincing data proving that it is the dimerization of ATP-synthase followed by oligomerization that engenders high local membrane curvature and promotes the formation of cristae

Presently, there are two alternative models of arrangement of respiratory supercomplexes and OXPHOS complexes in the inner mitochondrial membrane, which in fact are contemporary versions of the “solid” and “fluid” models, namely, a model of highly organized respiratory strings and patches (Nübel et al., 2009; Wittig, Schägger, 2009) and a “plasticity” model (Acín-Pérez et al., 2008; Enríquez, 2016), respectively. The first model describes the strings of respiratory supercomplexes associated with each other (see Fig. 1, d ), while the second postulates a random distribution of supercomplexes and complexes in the membrane (see Fig. 1, e). Moreover, both models assume separate location and autonomous functioning of respiratory supercomplexes and oligomeric rows of ATP-synthases.

Respiratory strings and patches

The first model is a development of the previously proposed model of respirasome (Schägger, Pfeiffer, 2000). On the basis of new data obtained by using BN-methods, H. Schägger and his colleagues (Nübel et al., 2009; Wittig, Schägger, 2009) put forward an assumption that respiratory supercomplexes in the inner mitochondrial membrane can be “building blocks” for larger structures, i. e. for respiratory strings and even for patches. Respiratory strings are linear rows of supercomplexes associated with each other in a certain order (see Fig. 1, d ). Depending on the organism and the species, either dimers or tetramers of complex IV may be the connecting links between the supercomplexes (Wittig, Schägger, 2009). The authors assumed that the respiratory strings can be spatially oriented parallel to each other in the membrane plane and interact via complex I monomers, while forming higher-order structures called “patches” (Nübel et al., 2009).

Identification (with the use of modified native gels with large pores) of multimeric respiratory supercomplexes with visible masses from 4–8 to 35–45 MDa was a convincing argument in favor of this model (Strecker et al., 2010). The authors also relied on the earlier pioneering work of R.D. Allen and his colleagues (Allen et al., 1989), who managed (with the aid of cryoelectron microscopy) to reveal not only oligomeric rows of ATP-synthases along the outer curve of tubular cristae of Paramecium multimicronucleatum but also additional rows of large particles along their inner curve, which were regularly arranged and corresponded in size to the dimeric complex I. H. Schägger and I. Wittig assumed that this additional group of projections represents a respiratory string and proposed a variant of such a string for mammalian mitochondria (see Fig. 1, d ) (Wittig, Schägger, 2009). Variants of respiratory strings for potato and Polytomella sp. were proposed by other investigators (Bultema et al., 2009; Miranda-Astudillo et al., 2018). At that, the respiratory strings were located parallel to the oligomeric rows of ATP-synthases (Miranda-Astudillo et al., 2018).

The “plasticity” model

The study of the composition of the OXPHOS system in various species with the aid of BN-PAGE revealed that – after solubilization of mitochondria with detergents – a part of the population of respiratory complexes remained in a free state, while another part was present in the supercomplexes (Enríquez, 2016). It was noted in a number of investigations that the relative amount of free and superassembled respiratory enzymes as well as the ratio of supercomplexes of diverse stoichiometry varied depending on the type of cells and the physiological state of the organism (stage of development, stress exposure, disease). On the basis of these data, J.A. Enríquez and his colleagues (Acín-Pérez et al., 2008; Acín-Pérez, Enríquez, 2014) proposed the “plasticity” model that postulated balanced coexistence of free respiratory complexes and supercomplexes of diverse composition and stoichiometry, the ratio of which corresponded to the physiological status of the cell (see Fig. 1, e). The proposed model was considered by the author mainly as a “refined revision of the fluid model” (Enríquez, 2016), since individual respiratory complexes and supercomplexes freely diffused in the plane of the inner mitochondrial membrane.

## The new subcompartmented oxphosomic model of organization of the phosphorylating system in mitochondria

According to the new model, a substantial part of the respiratory supercomplexes physically interacts with dimeric ATPsynthases, thereby forming oxphosomes, which are located mainly in the cristae subcompartment of mitochondria in the form of highly organized megastructures that are, in fact, “mini-factories” involved in ATP production (Ukolova et al., 2020) (see Fig. 1, f). At the same time, the rest of the respiratory complexes and supercomplexes obviously remain in free form. It is supposed that the ratio between the assembled oxphosomes and free respiratory supercomplexes and complexes depends on the type, physiological status and energetic needs of the cell. Actually, the model integrates the contemporary “solid” and “fluid” models (see Fig. 1, d, e), while adding a new layer of complexity related to the oxphosomic organization as well as to the structural and functional subdivision of the inner membrane into subcompartments.

Experimental data that contributed to the emergence of the model

**Fig. 2.**
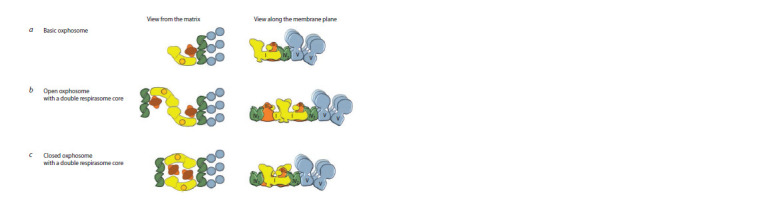
Putative organization of the basic oxphosome and oxphosomes with double respirasome core of open and closed types. The schematic view of putative oxphosomes from pee shoot mitochondria is shown. The view from the matrix side of the mitochondrial inner membrane and the side-view (along the membrane plane, prof ile projection) are shown. The color code is the same as in Fig. 1.

As noted above, the model was developed on the basis of our data obtained recently by analysis of mitochondria from etiolated pea shoots (Ukolova et al., 2020). The usage of freshly isolated organelles for digitonin solubilization of OXPHOS supercomplexes and complexes, application of multimeric electrophoresis system based on BN-PAGE, and mild electrophoretic separation conditions made it possible to identify supercomplex IV1Va2 and demonstrate the possibility of physical interaction between ATP-synthase and respiratory complex IV. Furthermore, in addition to the canonical associations I1III2, I1III2IVn and III2IV1–2, dimer V2 and free complexes I–V, we were able to reveal new structures that had not been not detected earlier, i. e. the second form of ATPsynthase Va having a higher molecular weight, respirasome I2III4IVn with two copies of complex I and double dimer III2, as well as a megacomplex (IIxIIIyIVz)n of high molecular weight. Simultaneous isolation of supercomplex IV1Va2, respirasomes I1–2III2–4IVn and megacomplex (IIxIIIyIVz)n, in which complex IV was bound up either with ATP-synthase or with respiratory complexes, allowed us to suppose that all the OXPHOS complexes in vivo can physically interact with a definite stoichiometry to form a larger structure that may be called an “oxphosome”. Thus, putative oxphosome represents a structure, in which complexes I (and/or, possibly, II), III2, IV and V are associated in strictly definite stoichiometry, and which can autonomously fulfill the whole cycle of reactions from the substrate oxidation to ADP phosphorylation, i. e. may “breathe” and produce ATP (Fig. 2).

It is possible to suppose that the connecting link between the respiratory and the phosphorylating parts of the oxphosome are dimers or tetramers of complex IV (see Fig. 2). However, the potential ability of complex IV to bind only respiratory supercomplexes to each other (via the formation of dimers and tetramers) has already been considered earlier in models of respiratory strings (Bultema et al., 2009; Wittig, Schägger, 2009; Miranda-Astudillo et al., 2018). Taking into account high abundance of free forms of complex IV (IVa/b and IV2) after solubilization with a detergent, both in our work (Ukolova et al., 2020) and in other works (Eubel et al., 2003; Krause et al., 2004; Acín-Pérez et al., 2008), it may be assumed that this connecting link is detergent-sensitive and represents the break point of the oxphosome in course of solubilization. The dimers of complex V are also sensitive to detergent treatment and dissociate into monomers under these conditions, as it was shown for mitochondria of many species (Schägger, Pfeiffer, 2000; Eubel et al., 2003). Such sensitivity might explain the minor amount of the new supercomplex IV1Va2 in digitoninsolubilized mitochondria of pea shoots (Ukolova et al., 2020). Some indirect literary data indicate that similar associations exist in other species and organisms. For example, Z.H. Qiu et al. (Qiu et al., 1992) demonstrated the possibility of reconstructing the association of complexes IV–V in proteoliposomes from highly purified complexes IV and V isolated from the bovine heart. It was demonstrated in the investigations conducted on the yeast that the absence of dimer-specific ATP-synthase subunits in mutant strains (i. e. absence of ATPsynthase dimers) reduced the activity of complex IV and the rate of ATP synthesis, altered the kinetic control of complex IV over oxidative phosphorylation (Boyle et al., 1999), as well as affected the stability of supercomplex III2IV2 (Saddar et al., 2008). These facts allow one to assume that the oxphosomic organization of the phosphorylating system may have conservative features and be typical of many species, although, apparently, it also has to possess taxon-specific traits.

The assumed organization of oxphosomes

All the presently known supramolecular structures of the OXPHOS system have strictly defined stoichiometry and spatial organization. In order to determine the stoichiometry of oxphosomes and develop a model of their organization in vivo for a specific species or an organism, it is necessary to take into account (i) the quantitative ratio of the OXPHOS complexes and (ii) the available data on the structure and spatial organization of respiratory complexes and supercomplexes, as well as ATP-synthase dimers.

Analysis of the literary data has given evidence that the ratio of complexes can vary depending on organism, type and physiological status of the cell (Schägger, 2002; Dubinin et al., 2011; Peters et al., 2012). This means that the model of the OXPHOS system organization in each definite case may have specific features. Nevertheless, taking into account high conservation of the studied OXPHOS superstructures, it is possible to assume that the basic principles of the supramolecular arrangement of the system, i. e. the formation of oxphosomes, oxphosomic strings and free supercomplexes with definite stoichiometry and spatial architecture, shall be retained.

The exact ratio of complexes I:II:III:IV:V has been determined so far only for bovine heart mitochondria and is approximately 1:1.5:3:6:3 (Schägger, 2002). According to H. Schägger, at this overall stoichiometry of the OXPHOS complexes, for every 2 complexes I there are 3 complexes II, 3 dimers III2, 6 dimers IV2 (or 3 tetramers IV4) and 3 dimers V2. On the basis of these data, the respirasome model for bovine mitochondria, which considers associations only of respiratory complexes I, III, and IV, was developed. The model postulates that the “building blocks” for the network of mammalian respiratory chain complexes are the large supercomplex I1III2IV4 (presently known as respirasome) and the small supercomplex III2IV4, which exist in a 2:1 ratio in the inner membrane (see Fig. 1, c). The exact overall stoichiometry of the OXPHOS complexes for mitochondria of pea shoots has not yet been determined. Meanwhile, our data showed that 2/3 of complex III population represent a part of respirasomes (Ukolova et al., 2020), which is consistent with the model of H. Schägger. This made it possible to develop the preliminary model of the OXPHOS organization for pea shoot mitochondria based on the ratio given for bovine OXPHOS complexes (see Fig. 1, f ). Further investigation of abundance and the ratio of energy system’s enzymes in pea shoot organelles will make it possible to clarify the proposed model.

Thus, based on the data discussed above, it is possible to assume that the main structural components or “building blocks” of the OXPHOS system in the mitochondria of pea shoots are represented by: the basic oxphosome I1III2IV4V6, respirasome I1III2IV4 and supercomplex III2IV4. The basic oxphosome represents respirasome I1III2IV4 bound up with three ATP-synthase dimers (see Fig. 2, a). The second respirasome I1III2IV4 and supercomplex III2IV4 can also be bound up with the basic oxphosome, while forming higher order oligomeric structures (see Fig. 1, f and Fig. 2, b, c). Taking into account (i) the data of J.B. Bultema with co-authors (Bultema et al., 2009), who, using electron microscopy, have shown the presence of open and closed conformations of the supercomplex I1III2 from mitochondria of potato tubers, and (ii) our electrophoretic data indicating a difference in the structure of the two “heaviest” supercomplexes with the composition I2III4IVn, it is possible to assume the formation of “open” and “closed” oxphosomes with a double respirasome core (see Fig. 2, b, c). It is likely that these two forms can transform one into the other and, so, participate in regulation of the activity of the OXPHOS system. The small supercomplex III2IV4 can in vivo associate with complex II or alternative NAD(P)H dehydrogenases directing electrons from oxidizable substrates to complex III2 (see Fig. 1, f ). The spatial organization of the putative oxphosomes has been developed given the available cryoelectron microscopy data on the structure of individual OXPHOS complexes and supercomplexes in plant mitochondria (see Fig. 2).

Presently, the “degree of the energy system assembly” in pea shoot organelles is not clear. It is not clear as well what part of the inner membrane is occupied by oxphosomic suprastructures in mitochondria of this and other species and organisms. It may be assumed that the ratio and the composition of free and assembled (into oxphosomic patches) respiratory supercomplexes and complexes may depend on the physiological status and energy requirements of the cell. For example, in cells with higher needs for energy (cells of muscles, heart, brain of mammals), it is logical to expect a higher abundance of assembled oxphosomic patches, oriented to production of large amount of ATP.

Subcompartmental localization of OXPHOS components

The inner mitochondrial membrane is subdivided into two morphologically and presumably functionally distinct subcompartments: the cristae and the inner boundary membrane domain. It has been shown that OXPHOS complexes are predominantly localized in the cristae domain (Gilkerson et al., 2003; Vogel et al., 2006). So, according to the data of R. Gilkerson with co-authors (Gilkerson et al., 2003), about 94 % of complex III of the respiratory chain and ATP-synthase is localized in the cristae, and only 6 % – in the inner boundary membrane. On the basis of available literary data, it may be assumed that the oxphosomes are predominantly located in the cristae domain, where they can form highly organized oxphosomic strings or patches (see Fig. 1, f ). At the same time, data of F. Vogel with co-authors (Vogel et al., 2006) showed that dimers of ATP-synthase were also present in the inner boundary membrane, which allowed one to assume the formation of oxphosomes in this subcompartment as well. Oxphosomes and individual supercomplexes located in the inner boundary membrane could efficiently support the potential-dependent and ATP-dependent processes that are most active in this domain, for example, protein translocation and assembly.

## The facts conf irming the functional validity of the oxphosomic model

The first hypothesis proposed the mechanism of proton coupling of oxidation and phosphorylation in a “rigidly” fixed assembly of OXPHOS enzymes, which later received experimental confirmation, was the hypothesis of local coupling proposed by R.J. Williams in 1961 (Williams, 1961). The hypothesis stated that such a spatially fixed organization of enzymes creates the conditions for the formation of protons in a high local concentration and for their direct (without crossing the hydrophobic membrane barrier) transfer to ATP-synthase (Williams, 1961; Skulachev, 1982). It is curious that the conception of R.J. Williams was proposed in the same year as P. Mitchell’s chemiosmotic hypothesis (which later became a theory), and was an alternative to the latter. The hypothesis of P. Mitchell (Mitchell, 1961) postulated that protons H+ are transported by proton pumps through the inner mitochondrial membrane into the water phase and do not bind to the membrane, while forming a delocalized electrochemical potential, which is used by ATP-synthase (Skulachev, 1982). Later, this mechanism was interpreted in favor of free (dissociated) distribution of enzymes in the membrane, that is, in favor of the “fluid” model.

Despite the huge amount of experimental data confirming the chemiosmotic hypothesis, the data supporting the concept of local coupling, and, consequently, the physical between mitochondrial ATP-synthase and complexes of the respiratory chain was shown (Tu et al., 1981; Krasinskaya et al., 1984); (2) evidence was obtained for the existence of a non-equilibrium membrane-bound fraction of hydrogen ions forming locally as a result of functioning of proton pumps in the respiratory chain (Antonenko et al., 1993; Motovilov et al., 2009); (3) participation of the fraction of membrane-bound protons in ATP synthesis was revealed (Solodovnikova et al., 2004; Eremeev, Yaguzhinsky, 2015); (4) catalysts of release of membrane-bound protons from the outer surface of the inner membrane were revealed, with their help the possibility of switching the phosphorylating system from the local coupling mode to the transmembrane proton transfer mode was shown (Yaguzhinsky et al., 2006). These data support the oxphosomic hypothesis and suggest that the OXPHOS system can use both localized and delocalized electrochemical potentials of hydrogen ions.

## Conclusion

New discoveries concerning organization, structure and functioning of the phosphorylating system of mitochondria periodically induce a revision of existing conceptions and initiate transition to a new level in understanding of the system’s arrangement and functioning. Emerging models either completely disprove the previous ones, as was the case in the late 1970s – early 1980s when the “solid” model was replaced with the “fluid” one (see Fig. 1, a, b), or are based on the previous ones, expanding and developing them, as, for example, in case of the model of respiratory strings, which developed the respirasome model (see Fig. 1, c, d ).

Presently, despite the active acquisition of data on spatial organization, structure and functional activity of respiratory supercomplexes and oligomeric ATP-synthase, the supramolecular organization of the OXPHOS system in vivo remains unclear. The latest data obtained in our investigations on mitochondria of etiolated pea shoots gave evidence of the possibility of physical association between respiratory supercomplexes and dimeric ATP-synthase (Ukolova et al., 2020). This information led to revision of the existing contemporary ideas of organization of the OXPHOS system (Wittig, Schägger, 2009; Acín-Pérez, Enríquez, 2014; Miranda-Astudillo et al., 2018) and to the proposal of the subcompartmented oxphosomic model (see Fig. 1, f ).

This model presupposes the existence in vivo of highly organized associations between the oligomeric rows of ATPsynthase and respiratory supercomplexes, which represent oxphosomic strings or patches that are localized predominantly in the cristae subcompartment. It is assumed that the oxphosomic organization allows a significant increase in the functionality of the OXPHOS system and efficiency of its operation. Firstly, association of oligomeric ATP-synthase with respiratory supercomplexes could enable the system to use not only protons in the bulky water phase of the intermembrane space, but also membrane-bound protons forming in high local concentrations as a result of operation of the assembled respiratory enzymes. Secondly, such an organization could involve additional layers of the system’s activity regulation (for example, transforming an open, possibly more active, form of oxphosome to a closed one, or changing the ratio of associated with ATP-synthases and free supercomplexes). Thirdly, the “anchorage” of the respiratory supercomplexes on the oligomeric rows of ATPsynthase could determine the morphology of the membrane to some extent. Further investigations will make it possible to clarify and probably visualize the supramolecular structure of the OXPHOS system in mitochondria of various organisms in vivo.

## Conflict of interest

The authors declare no conflict of interest.
